# Metagenomic insight into the microbial networks and metabolic mechanism in anaerobic digesters for food waste by incorporating activated carbon

**DOI:** 10.1038/s41598-017-11826-5

**Published:** 2017-09-12

**Authors:** Jingxin Zhang, Liwei Mao, Le Zhang, Kai-Chee Loh, Yanjun Dai, Yen Wah Tong

**Affiliations:** 1 Environmental Research Institute, National University of Singapore, 1 Create Way, Singapore, 138602 Singapore; 2 Department of Chemical & Biomolecular Engineering, National University of Singapore, 4 Engineering Drive 4, Singapore, 117576 Singapore; 3 School of Mechanical Engineering, Shanghai Jiao Tong University, 800 Dong Chuan Road, Shanghai, 200240 China

## Abstract

Powdered activated carbon (AC) is commonly used as an effective additive to enhance anaerobic digestion (AD), but little is known about how the metabolic pathways resulting from adding AC change the microbial association network and enhance food waste treatment. In this work, the use of AC in an anaerobic digestion system for food waste was explored. Using bioinformatics analysis, taxonomic trees and the KEGG pathway analysis, changes in microbial network and biometabolic pathways were tracked. The overall effect of these changes were used to explain and validate improved digestion performance. The results showed that AC accelerated the decomposition of edible oil in food waste, enhancing the conversion of food waste to methane with the optimized dosage of 12 g AC per reactor. Specifically, when AC was added, the proponoate metabolic pathway that converts propanoic acid to acetic acid became more prominent, as measured by 16S rRNA in the microbial community. The other two metabolic pathways, Lipid Metabolism and Methane Metabolism, were also enhanced. Bioinformatics analysis revealed that AC promoted the proliferation of syntrophic microorganisms such as *Methanosaeta* and *Geobacter*, forming a highly intensive syntrophic microbial network.

## Introduction

The increasing food waste (FW) generation around the world has increased the demand for reduction and effective utilization of FW in recent years^1^. The uncontrolled disposal of FW is liable to create public health concerns and cause adverse environmental impacts. Anaerobic digestion (AD) has become a proven and promising approach for FW treatment and bioenergy production^2, 3^. During AD process, biodegradable organic matters can be converted into biogas (CH_4_ and CO_2_) via anaerobic microorganisms. However, AD of FW still faces challenges in process stability and effectiveness under high organic loading rates (OLRs) during AD process^4^.

AD is a complex biological process, which contains many different consortia of microorganisms with different functions^5^. Specifically, hydrolyzing and fermenting bacteria initially convert complex organic compounds into volatile fatty acids (VFA), alcohols and lactate. These intermediate products are further converted by fermentative bacteria to acetate, H_2_, CO_2_, and formate that are used by methanogens for acetoclastic and hydrogenotrophic methanogenesis. Traditional H_2_-utilizing methanogens can generate methane by using fermentative end-products such as H_2_/CO_2_ deriving from the syntrophic metabolism of syntrophic bacteria. The relationship between syntrophic bacteria and H_2_-utilizing methanogens is syntrophic and this process is well-documented interspecies hydrogen transfer (IHT)^6^. The activity of syntrophic bacteria is liable to be affected by the hydrogen concentration in the liquid phase of AD^7^. Under high OLRs, if the removing rate of hydrogen by methanogens is relatively low, the thermodynamics of IHT process will be inhibited, resulting in the accumulation of VFA, pH decline and failure of AD operation.

Enhancing AD by the addition of conductive carbon materials in digesters to resist high OLRs has been recently reported^8^. Further studies showed that direct interspecies electron transfer (DIET) has been considered as an alternative to IHT for syntrophic metabolism^9^. This process can be explained as the mechanism of direct electron transfer from the bacteria to the electron-accepting methanogens via biological electrical connections, e.g. electrically conductive pili^10^ and outer surface c-type cytochromes^11^. However, most of these studies have been conducted to explore only the potential mechanisms using simple substrates^9^ or pure cultures^10^ due to the complexity of the degradation process of complex organic substrates in AD process^12^, thus limiting the exploration of metabolic mechanism. For FW, in addition to syntrophic metabolism of AD intermediate products via IHT or DIET by syntrophic bacteria, hydrolyzing and fermenting process via other bacteria also plays an important role in AD process^13^. For example, the hydrolysis and acidogenesis of lipids is considered as a critical limiting step for AD of FW due to its bio-refractory and hydrophobic property^14, 15^. Therefore, syntrophic metabolism is unlikely to work effectively unless hydrolysis and acidogenesis function well. However, the potential microbial networks and metabolic pathways at the whole community level have yet to be investigated, especially for AD of FW.

Based on the above consideration, adding AC in AD system was investigated for FW treatment and methane production. Deep insight into the effects of AC on the interactive relationship in a broad profile of microbial networks including hydrolyzing and fermenting bacteria, syntrophic bacteria, and methanogens was conducted by the metagenomic shotgun sequencing using high-throughput sequencing technology, which typically generates millions to billions of reads for the metagenomic DNA extracted from sludge samples of AD reactors. In addition, the effects of AC on the syntrophic mechanism and metabolic pathway of all microbes were explored by a method of metabolic pathway analysis that provided systematic information about gene function, protein function and enzymatic reaction based on the database of Kyoto Encyclopedia of Genes and Genomes. To the best of our knowledge, this is the first study to conduct multi-analysis regarding the taxonomic resolution of microbial communities and metabolic pathway of anaerobic microbes as well as association analysis of microbial network in AD process assisted by the addition of AC.

## Results and Discussion

### Effect of AC addition on methane production in AD of FW

**Figure 1:**
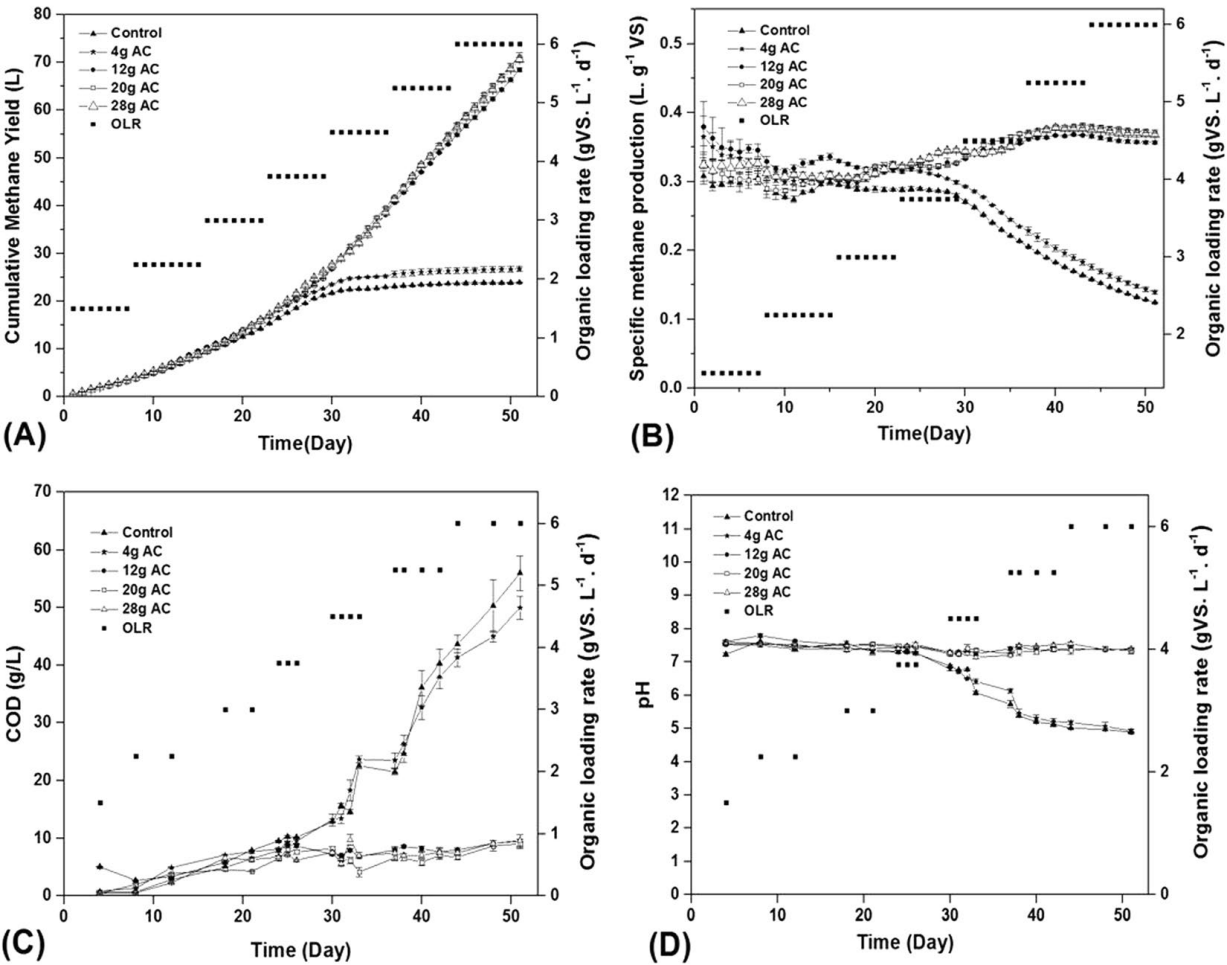
Effect of different dosages of AC on (**A**) Cumulative methane yield, (**B**) Specific methane production, (**C**) COD concentration and (**D**) pH.

The effect of AC addition on methane production was investigated in five AD reactors operated in parallel: four AC enhanced AD reactors with the addition of different content of AC: 4 g, 12 g, 20 g, and 28 g and control AD reactors without the addition of AC. Cumulative methane yield (CMY) (Fig. 1A), specific methane production (SMP) (Fig. 1B), COD (Fig. 1C), and pH (Fig. 1D) were monitored at increasing OLR from 1.5 to 6 g VS·L^−1^·d^−1^.

In the initial 22 days of OLR up to 3 g VS·L^−1^·d^−1^, the average CMY and SMP in the five AD reactors were increased to 15.6 L and 0.32 L.g^−1^ VS, respectively, and all reactors operated stably with an average effluent pH of 7.4. Differences among the five AD reactors were hardly significant due to the low OLR. When the OLR was increased to 6 g VS·L^−1^·d^−1^, the CMY in AD reactors with the addition of AC greater than or equal to 12 g was maintained between 68.5 L and 71.2 L and SMP was kept between 0.36 L.g^−1^ VS and 0.37 L.g^−1^ VS with an average effluent pH of 7.3. The CMY was only 24.2 L and 22.3 L in the AD reactor with the addition of 4 g AC and control reactor, respectively, and the SMP had dropped to 0.14 L.g^−1^ VS and 0.12 L.g^−1^ VS, respectively, which might be caused by the high COD concentration of 50 to 56 g·L^−1^ and low pH of 5 to 6. These results indicate that 12 g AC is the optimal additive amount to tolerate high FW loading rates and promote methane production as increase in AC dosage cannot further improve AMY and SMP significantly, while AC dosage lower than 12 g has not positive effect on the enhancement of AD performance for methane production. This phenomenon can be explained by the abundance of archaea which comprise the majority of methanogens in AD reactors. From Fig. S1, 16S rRNA gene copy numbers of archaea in the sludge samples of AD reactors with 12 g AC was 2.93 × 10^6^ copies/μL – DNA, significantly higher than that of 5.29 × 10^5^ copies/μL – DNA in the AD reactors with 4 g AC. However, 16S rRNA gene copy numbers of archaea in AD reactors with 20 g AC and 24 g AC were only 3.16 × 10^6^ copies/μL - DNA and 3.09 × 10^6^ copies/μL – DNA, respectively, presenting no significant difference as compared with the AD reactors with 12 g AC.

AC is an amorphous, carbonaceous material exhibiting relatively high porosity, large surface area, and strong adsorption capability. Therefore, the surface of AC in the four AD reactors could provide an excellent environment for colonization by anaerobes, especially for methanogens, which likely helped the reactor operate stably. Too low amount of AC might reduce the amount of active sites of AC^16^ and affect the enrichment of methanogens in the AD reactor^16^, a possible reason to explain the poor performance of the AD reactor with the addition of 4 g AC. However, excessive dosage of AC also cannot further enhance the enrichment of methanogens and improve reactor performance.

### Microbial community composition

**Figure 2:**
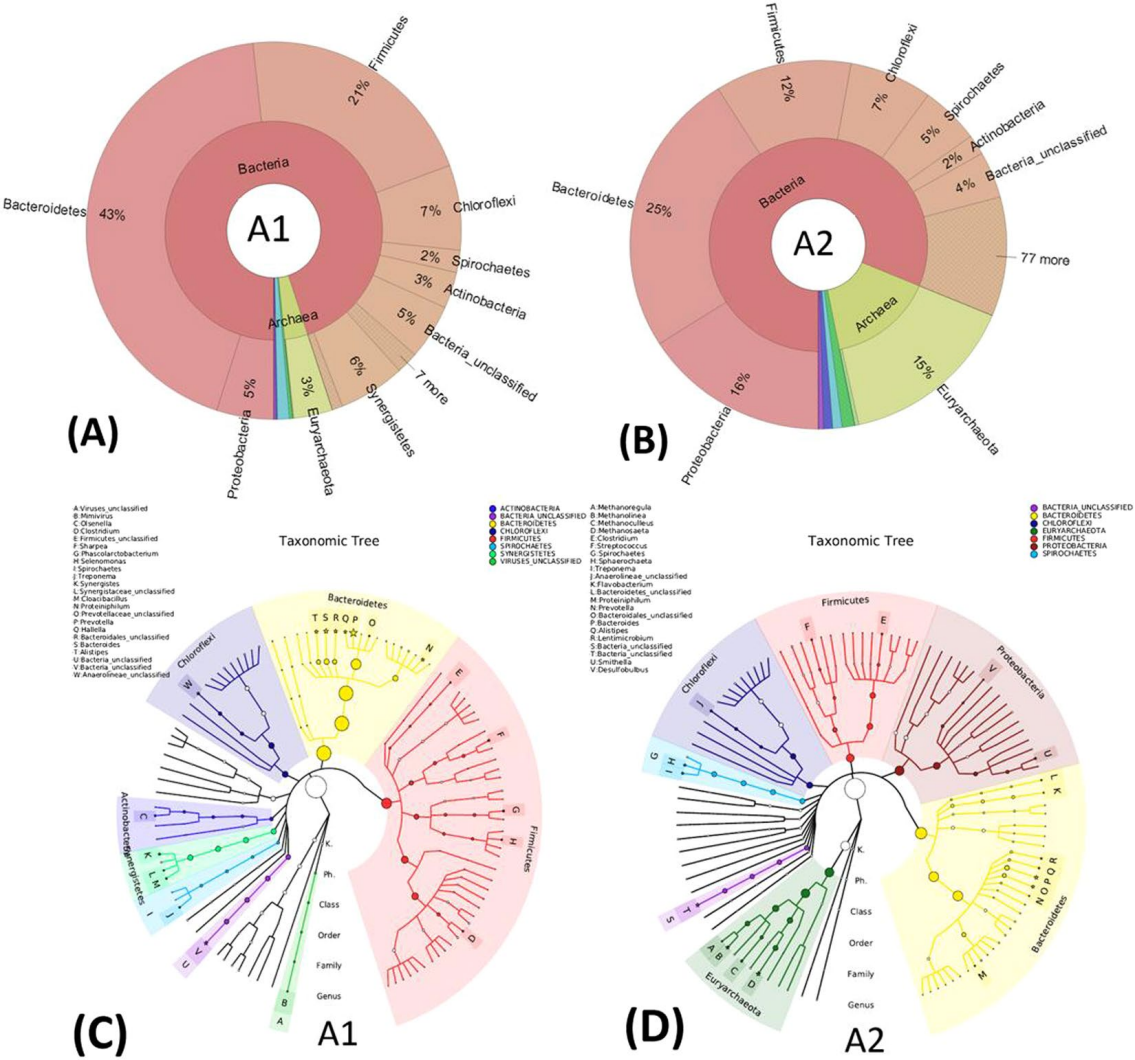
Schematic diagram of multi-level species taxonomy of the whole AD microbial community in the sludge samples of A1 (**A**) and A2 (**B**) after 51 days of operation. Microbial community compositions from the interior to external circle were identified at domain and phylum level by analysing merged paired-end based16s rRNA genes. Phylogenetic and taxonomy trees of dominant AD microbial communities in reactor A1 (**C**) and A2 (**D**) according to GraPhlAn analysis. The top one hundred genus were selected to construct phylogenetic trees, and the corresponding phyla of top twenty genus (marked with asterisk) was marked in different color. The size of circles and asterisks represent the different relative abundance of microbial populations.

After 51 days of operation, the whole microbial community in the sludge samples of seed sludge, control reactor without AC (A1), and the AD reactor with the addition of 12 g AC (A2) were analyzed by metagenomic shotgun sequencing. Multiple species taxonomy analysis showed that bacterial and archaeal populations are dominant microbial communities, accounting for over 97% of total microbial populations (Fig. 2A,B). The remaining populations are *Metazoa*, Unclassified Viruses, Fungi, Viridiplantae, and Plasmodium_chabaudi, revealing just a tiny fraction of the whole microbial communities. Hierarchical cluster analysis (Fig. S2) showed that the microbial community in seed sludge and A2 was classified to one cluster. This cluster was separated from A1, suggesting that the microbial community structure in A1 significantly changed without the addition of AC.

Error bar graphs of difference comparison in Fig. S3 further indicated that the difference of microbial communities between A2 and A1 and seed sludge was mainly attributed to the difference between proportions (p < 0.05) of *Proteobacteria*, *Bacteroidetes*, and *Euryarchaeota*. *Proteobacteria* and *Bacteroidetes* include fermenting, acetogenic and syntrophic bacteria that can ferment organic compounds and degrade VFA^17^. As shown in the outer ring of cyclic phylogenetic trees in Fig. 2C,D, two dominant genera (*Smithella*, *Desulfobulbus*) under *Proteobacteria* and two dominant genera (*Flavobacterium* and *Lentimicrobium*) under *Bacteroidetes* in bacterial community were selectively enriched in A2 due to the addition of AC. The relative abundance of *Proteobacteria* in A2 were 16% of total microbial communities while it was only 5% in A1. *Desulfobulbus*, found with the ability of reducing sulfate to hydrogen sulfide, is also involved in the production of propionate and acetate via fermenting complex organic compounds^18^. *Smithella* was the typical syntrophic VFA oxidizing bacteria known to degrade butyrate and/or propionate to acetate with the production of H_2_. This genera usually has syntrophic relationship with H_2_-utilizing methanogens via IHT. Most of genera in *Bacteroidetes* are hydrolyzing and fermenting bacteria^19^, such as dominant genera *Flavobacterium* and *Lentimicrobiu*, as well as other genera such as *Bacteroidetes* and *Alistipes*. These genera are involved in VFA, CO_2_ and H_2_ generation, through carbohydrates, lipids and proteins fermentation producing enzymes for polysaccharides’ and proteoglycans’ cleavage^19, 20^.

*Euryarchaeota* comprise the majority of methanogens in AD reactors. From Fig. 2A,B, over 93% of anaerobes in phylum *Euryarchaeota* belong to methanogens except *Thermoplasmata*, *Thermococcales*, and *Halobacteria*. The relative abundance of *Euryarchaeota* in A2 is 15% of total microbial communities, significantly higher than the 4% in seed sludge and 3% in A1. These results indicated that the addition of AC in A2 helped to enrich methanogens, playing a crucial role in improving methane yield during AD process. On the basis of metabolic pathway, methanogens can be categorized into aceticlastic methanogens and hydrogen-utilizing methanogens. As shown in the outer ring of cyclic phylogenetic trees in Fig. 2C,D, four dominant methanogenic archaea (A, B, C, D), three hydrogenotrophic methanogens (*Methanoregula*, *Methanolinea*, *Methanoculleus*), and one aceticlastic methanogen (*Methanosaeta*) were only enriched in A2. Even through A1 also has 3% of *Euryarchaeota*, the relative abundance of each genera is lower than 1% of total microbial communities. In the sludge samples of A2, *Methanosaeta* accounted for 5% of total microbial communities. As is known, *Methanosaeta* is capable of directly accepting electron from the reduction of CO_2_ to produce CH_4_ through participating DIET^10^. However, DIET has only been reported to occur in defined co-cultures of *Geobacter Metallireducens* and *Geobacter sulfurreducens* or *Geobacter* species and acetoclastic methanogens^10, 21^. *Geobacter* is included in *Proteobacteria*. As shown in Fig. S4, the relative abundance of *Geobacter* genera in A2 was 0.8% of bacterial communities, much higher than the 0.08% in A1 and 0.3% in seed sludge, respectively. *Geobacter metallireducens* and *Geobacter sulfurreducens* are two dominant *Geobacter* species in A2 accounting for 25% of total *Geobacter* species. Together with the increase of *Methanosaeta* genera and *Geobacter* genera, the potential DIET between *Geobacter* and *Methanosaeta* might be established to improve the syntrophic metabolism of AD intermediate products e.g. propionate, butyrate, and ethanol. Therefore, the contribution of DIET on AD of FW in A2 might be enhanced via the addition of AC. Furthermore, the dominant hydrogenotrophic methanogens, *Methanoregula*, *Methanolinea* and *Methanoculleus*, accounted for 41% of archaeal communities. The enrichment of hydrogenotrophic methanogens play a major role in keeping a low H_2_ pressure through reduction of CO_2_/H_2_ to CH_4_ and has been found to be of importance for syntrophic metabolism of VFA to acetate by syntrophic oxidizing bacteria^22^.

### Microbial network and co-network

**Figure 3:**
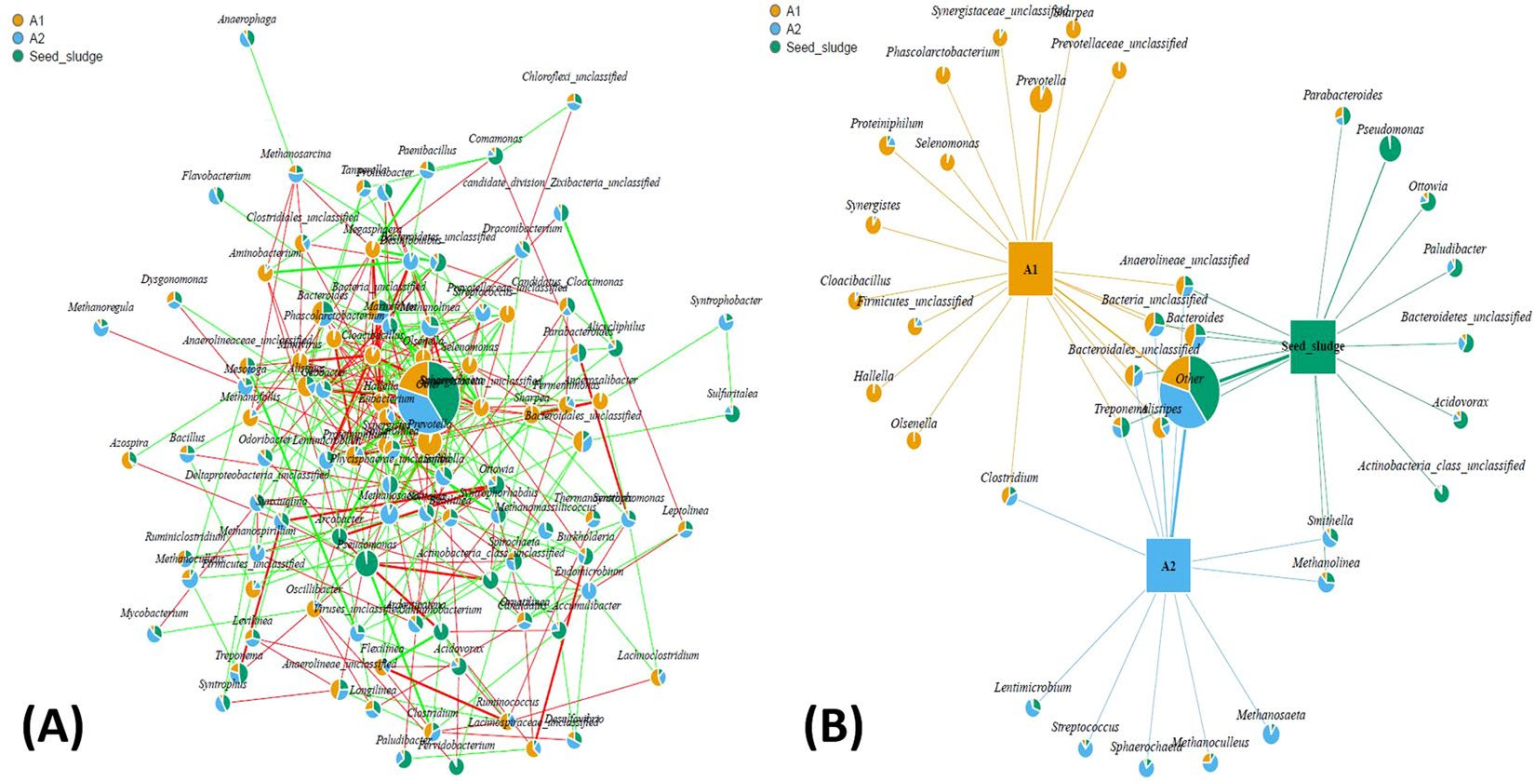
(**A**) The microbial co-network revealing intense interaction between different genus in all the sludge samples of reactor A1, A2 and seed sludge. Pie charts indicate the relative abundance of each genus. The red line represent the positive associations, while the green line represent the negative associations. (**B**) The microbial network indicating the shared and unique dominant genus among the seed sludge, reactor A1, and A2.

The AD process generally requires multiple groups of microorganisms working together to convert organic substrates to methane. Therefore, to investigate a thorough microbial network in relation to the whole microbial community changes, understanding the relationship among different microorganisms is very important. In particular, analyzing the impact of AC on the microbial communities is crucial. As shown in Fig. 3A, a microbial co-network was constructed to present correlations among all microorganisms in A1, A2, and seed sludge. The area of all pie charts is similar except the pie of the “Other” is composed by several non-dominant microorganisms, suggesting that the difference of relative abundance of every microbial genera is hardly significant. This result might be partially attributed to the complexity of the substrate and degradation process that usually requires the joint action of multiple genera, as compared with simple substrate such as acetate that might only need a limiting amount of genera. The larger area of pie “Other” indicated that the function of some non-dominant microorganisms is also indispensable even though dominant microorganisms usually play a major role in AD process. The red line and green line respected the positive and negative correlations among different genera, respectively. Positive correlations usually contain cross-feeding, co-aggregation, co-colonization and niche overlap^23^, which are favorable for the syntrophic metabolism of AD intermediates among various genera. In AD conditions, negative correlations usually arose from competition for substrates and differential niche adaptation. The distribution of most genera is intensive except the genera locating at the external border, indicating that some genera has little relationship with AD process such as *Viruses_unclassified*, *Azospira* and *Mesotoga* etc. while some genera are inseparable from AD process e.g. *Bacteroides* and *Methanosaeta*.

To further investigate the effects of AC on the interaction between different genera, a thorough microbial network among A1, A2 and seed sludge was constructed with dominant microbial populations that has significant difference (P < 0.05) among each genera (Fig. 3B). This microbial network revealed topological features observed in complex systems, forming shared correlations and specific correlations. The main shared correlations is attributed to the same microorganisms existing within both A1 and A2 reactors, and within seed sludge e.g. *Bacteroides*, *Treponemalistipes*, *Anaerolineae_unclassified*, and others. These genera are involved in fermenting complex organic compounds to simple substrates such as VFA, CO_2_ and H_2_. One possible explanation for the aggregation of these genera in the shared area is that hydrolysis and fermentation are the first steps in AD process via the action of hydrolyzing and fermenting bacteria that can survive against variable environment. However, the addition of AC in A2 changed the AD environment and reshaped the microbial network by forming some specific correlations that were attributed to the specific genera which only survive in A2 e.g. *Lentimicrobium*, *Streptococcus*, *Sphaerochaeta*, *Methanoculleus*, and *methanosaeta*. *Methanoculleus* and *Methanosaeta* are classified as hydrogenotrophic methanogen and aceticlastic methanogen, respectively. *Lentimicrobium*, *Streptococcus* and *Sphaerochaeta* are strictly fermentative bacteria which produce VFA, ethanol, H_2_/CO_2_ by fermenting complex organics.

### Metabolic pathways of microbial communities

**Figure 4:**
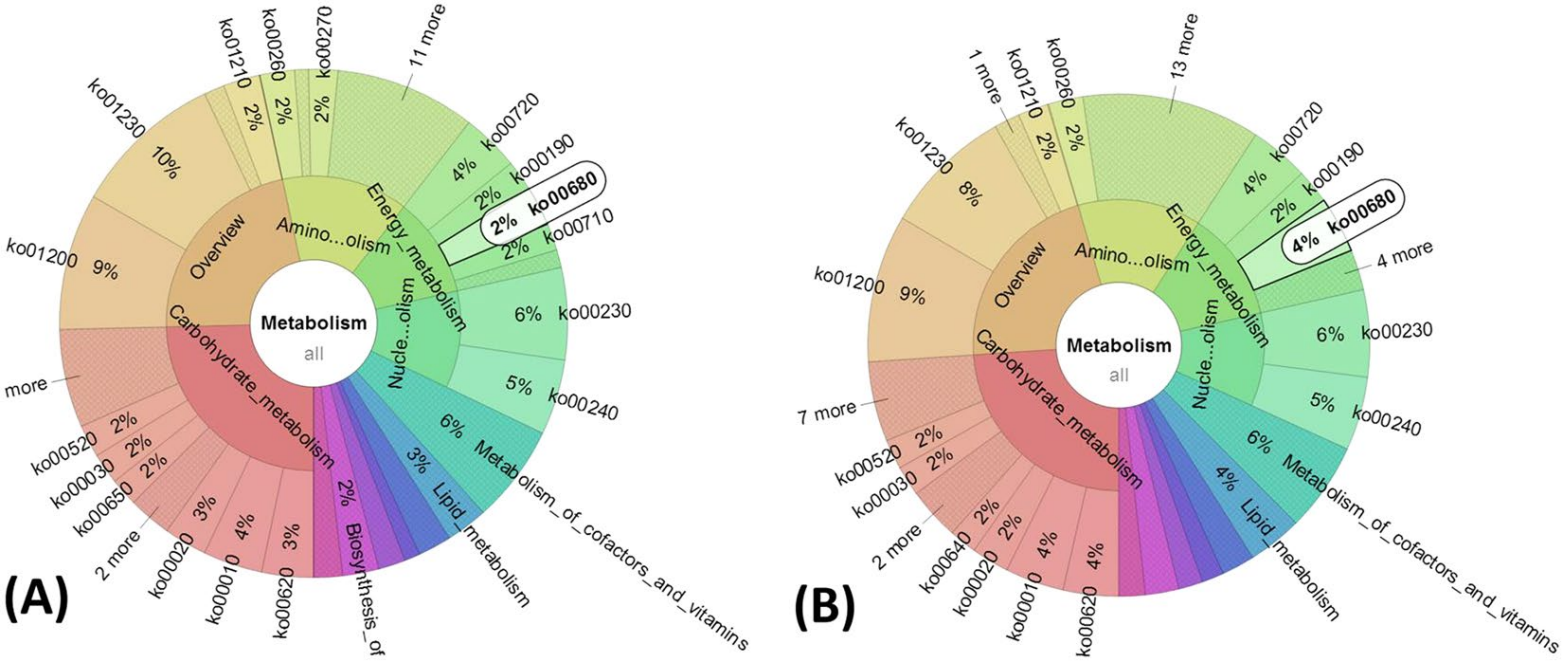
Metabolic pathways of microbial communities, according to gene KEGG pathway analysis. (**A**,**B**) Functional profiling of the whole microbial community in the category of “Metabolism” in A1 and A2, respectively. White pie charts indicate the relative abundances of gene ko00680 in the category of “Metabolism”.

To understand and exploit the impact of AC on microbial metabolism, the analysis of metabolic pathways of microbial communities was conducted. The predictive functional profiling of the microbial communities in A1 and A2 was shown in Fig. S5. All the metabolic functions were classified to Metabolism, Environmental_Information_Processing, Genetic_information_Processing, Cellular_Processes, Human_Diseases, and Organismal_Systems, of which Metabolism was the most dominant category that included several metabolic pathways e.g. Carbohydrate Metabolism, Amino Acid_Metabolism, Lipid Metabolism, Energy Metabolism, and Others. These metabolic pathways in Metabolism denotes the various biochemical processes responsible for the breakdown and mineralization of carbohydrate, proteins, and lipids in FW and bioenergy production. However, the differences of metabolic pathways between A1 and A2 were hardly significant except those of Energy Metabolism and Lipid Metabolism. Both of them were improved by 2% and 1%, respectively, in relative abundance of whole genome, due to the addition of AC in A2. To further verify the metabolic functions of microbial communities, analysis of gene functional classification were also conducted through comparing protein sequence with another database of COG (https://www.ncbi.nlm.nih.gov/COG/). As shown in Fig. S11, the gene numbers of Energy production and conversion and Lipid transport and metabolism in A2 were 15030 and 5395, respectively, while it were only 13863 and 4825 in A1, respectively. The enhancement of Energy Metabolism was mainly attributed to the pathway of Methane Metabolism (Ko00680) (Fig. 4), in which methanogens obtain energy for growth by converting simple substrates to methane during AD process (Fig. S6A). This might be a possible biological reason for the higher methane yield in A2.

Lipid Metabolism refers to the degradation of lipids. The improvement of Lipid Metabolism was ascribed to the pathway of Fatty Acids Degradation, in which hydrolyzing and fermenting bacteria converted long-chain fatty acids and glycerolipid to small molecule fatty acids via the action of several functional enzymes (Fig. S6B). The degradation of lipids is still considered to be the limiting step for AD of FW because of the bio-refractory compounds contained in lipids that mainly include fats and edible oil^14^. Moreover, the hydrophobic feature of lipids is liable to affect the mixing of lipids and microorganisms, influencing the degradation of lipids in AD process. Considering the absorptive ability, AC could be potentially used as an effective additive for enhancing the metabolism of lipids by simultaneously absorbing lipids or oil components and facilitating microorganism colonization, and subsequently accelerating the degradation rata of lipids by microorganisms. To confirm this, bench-scale experiments were conducted using edible oil as a single substrate to investigate the effect of AC on AD of lipids. Figure S7 shows the variations of methane yield in AD reactors with the addition of AC (ACR) and control reactors without the addition of AC (CR). These two AD reactors operated in parallel at increasing OLR from 1 to 6 g oil · d^−1^. At an OLR of 1 g oil · d^−1^, the average methane yield in the two reactors varied between 58 and 62 ml · d^−1^, and pH ranged from 7.0 to 7.2. These differences were hardly significant. With further increase in OLR, the average methane yield in ACR increased monotonically to 325 ml · d^−1^ at OLR of 6 g oil · d^−1^ and the average pH was 7.3. The methane yield in CR was only 175 ml · d^−1^ and the average pH was maintained at 7.0. These results suggested that adding AC in AD process could enhance Lipid Metabolism, thus playing a major role in improving AD performance for FW treatment and methane production.

### Co-network of microbial metabolism and specific metabolic pathway in A2

**Figure 5:**
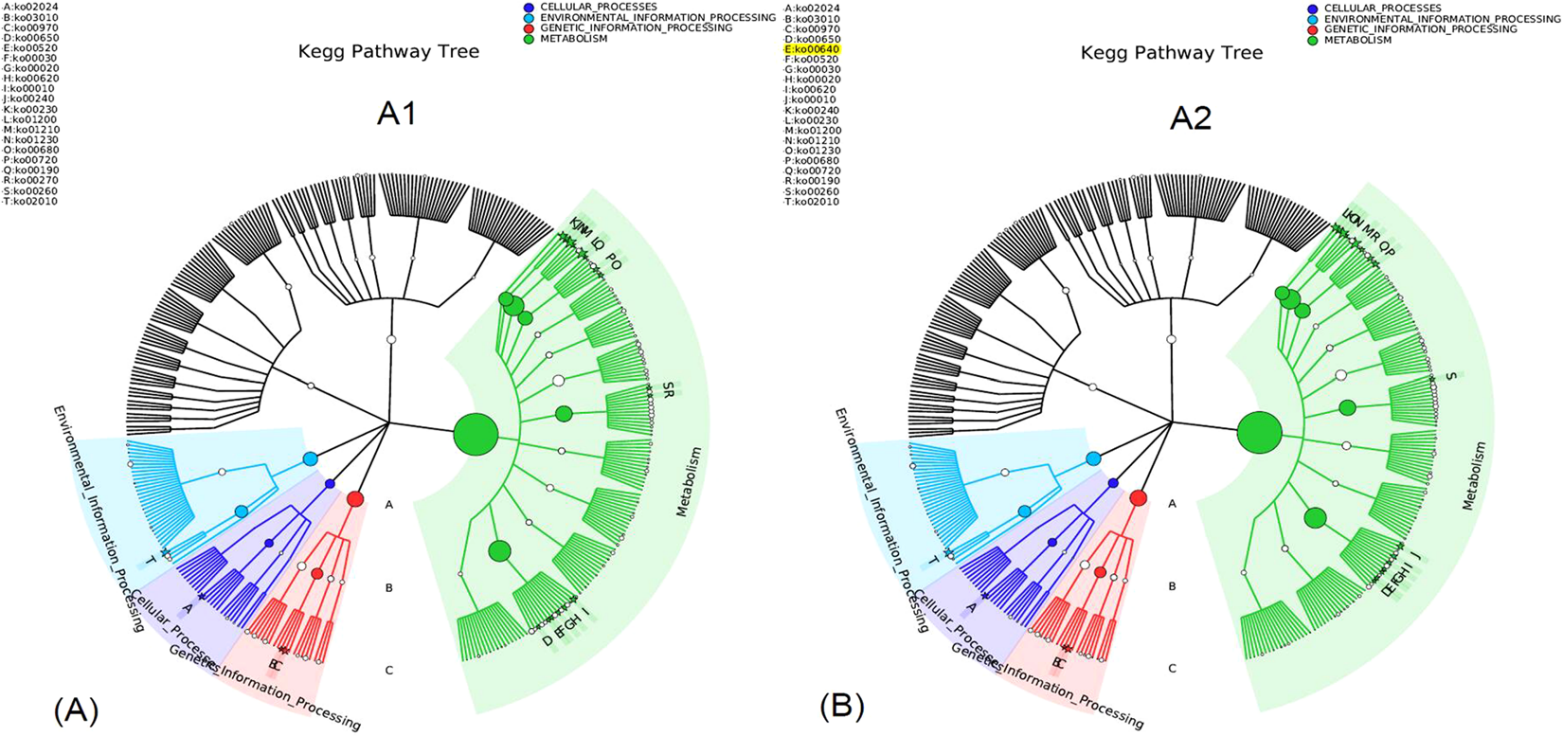
KEGG pathway trees represent the dominant metabolic pathways during AD process in reactor A1 and A2. Asterisks in the outer ring indicate the dominant metabolic pathways of reactor A1, A2. (**A**) reactor A1; (**B**) reactor A2.

Figure S8 shows the co-network of metabolic pathways of microbial communities in A1, A2, and seed sludge. On basis of the area of the pies, the metabolic pathways with the numbers of Ko01200 and Ko01230 are the most dominant metabolic pathways in all reactors. Ko01200 is the Carbon metabolism that contains carbon utilization pathways of glycolysis, some pathways of methane metabolism, and other carbon fixation pathways. Ko1230 is the biosynthesis pathway of amino acids for conversion of carbon-based compounds to amino acids. These two metabolic pathways are the most basic aspects in AD process for the degradation of organic compounds and methane production. The second dominant microbial metabolism related to the metabolic pathways of Ko00230, Ko02024, Ko02010, Ko00720, and Ko00620, corresponding to Purine metabolism, Quorum sensing, ABC transporters, Carbon fixation pathways, and Pyruvate metabolism, respectively. These results indicated that most of the functional metabolisms occur in the sludge samples of A1, A2, and seed sludge even when the operational conditions vary greatly. However, a specific metabolic pathway of Ko00640 was only founded in A2 (Fig. 5). This metabolic pathway is propanoate metabolism, in which propionate can be degraded by syntrophic bacteria to produce acetate and methanol as end-products (Fig. S9). This result was in agreement with the result of lower percentage of propionate (32%) in A2 as compared with the 43% in A1 (Fig. S10). The syntrophic acetogenic reaction of propionate to acetate is thermodynamically unfavorable due to high Gibbs free energy of +76.1 kJ/reaction, therefore propionate is liable to accumulate in AD system to cause the instability of AD operation. This could possibly explain the poor performance of A1. Together with the results of potential DIET by enriching *Geobacter* species and *Methanosaeta* (Figs 2 and S4) and low propionate in A2 (Fig. S10), the addition of AC in AD process might help to accelerate the syntrophic metabolism of propionate and further enhance AD performance.

### Significance of this work

**Figure 6:**
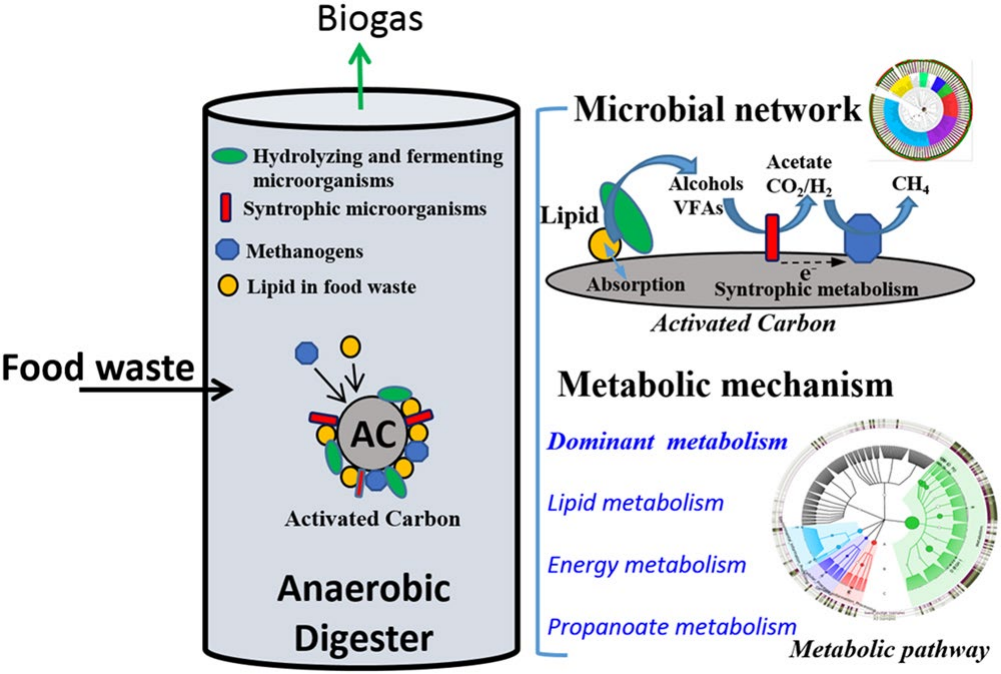
Conceptual graph of microbial network and dominant metabolic pathways of microbial communities in anaerobic digesters for food waste by incorporating AC.

A potential microbial synergy network was constructed and the dominant metabolic pathways of microbial communities in anaerobic digesters by incorporating AC were determined. A proposed conceptual graph of microbial network and dominant metabolic pathways was summarized in Fig. 6. The data revealed that AC accelerated the colonization of archaea, improving significant anaerobic performance for FW treatment and methane production. In agreement with the higher abundance of archaea and methane yield, the proof of metabolic pathway analysis found that the pathway of methane metabolism was enhanced simultaneously. Interestingly, AC also enhanced the degradation of edible oil and improved the pathway of lipid metabolism, thereby demonstrating a better performance of anaerobic digestion on food waste treatment. In agreement with most recent studies^8, 9, 12^, *Geobacter* species and *Methanosaeta* were significantly enriched by the addition of AC, thus suggesting that the potential DIET between *Geobacter* and *Methanosaeta* might be established to improve the syntrophic metabolism of AD intermediate products such as propionate. Importantly, further evidence of metabolic pathway analysis supported this speculation, since AC created a specific metabolic pathway of propanoate metabolism that accelerated the syntrophic metabolism of propionate. These findings demonstrate through investigating the 16S rRNA of the microbial community that the changes in microbial association network and biometabolic pathways explain how AC enhances AD of food waste.

## Conclusions

In this work, the use of AC in an anaerobic digestion system for food waste was explored. Using bioinformatics analysis, taxonomic trees and the KEGG Orthologs database, changes in microbial networks and biometabolic pathways were tracked.

1. 12 g AC in the reactor is the optimal additive amount to tolerate high FW loading rates.
2. Adding AC in wet AD system could enhance the metabolism of lipids in FW and facilitate archaea colonization, and subsequently accelerate the degradation rate of FW and methane production.
3. AC enhanced the biometabolic pathways of methane metabolism and lipid metabolism.
4. The addition of AC in AD process helped to construct an effective microbial network and accelerate the syntrophic metabolism. A specific metabolic pathway of propanoate metabolism was discovered by the addition of AC.

## Materials and Methods

### Inocula and substrates

The seed sludge was collected from a large-scale anaerobic digester at Ulu Pandan Water Reclamation Plant in Singapore. The ratio of volatile suspended sludge (VS) to total suspended sludge (TS) was 0.65 with an initial TS of 13.2 g/L. FW was obtained from a canteen of the National University of Singapore, which mainly consisted of rice, noodles, meat, vegetables, and condiments. After removing any bones and non-biodegradable waste like plastic bags, FW was homogenized by a blender and then stored in a −20 °C freezer. The detailed characteristics of FW are listed in Table S1.

### Reactor specification and operation

Two bench-scale experiments were conducted. First, four glass experimental anaerobic digesters were operated for FW treatment with the addition of different content of powdered AC (200 mesh): 4 g, 12 g, 20 g, and 28 g. The working volume of each digester was 0.8 L. Control digester is same to the experimental digesters but without the addition of powdered AC. The pore volume and surface area of powdered AC were 0.30 cc/g and 385 m^2^/g, respectively. After being seeded with seed sludge, these five anaerobic digesters were operated for AD of FW in a semi-continuous mode (feeding every day) with a gradual increase in the organic loading rate (OLR). All reactors were operated at 35 °C in parallel. The sludge retention time was 30 d. All the experiments were conducted in triplicate.

After getting the optimum dosage of AC for AD of FW, two glass experimental anaerobic digesters were operated for the treatment of edible oil of food waste with the addition of 12 g AC (hereafter referred to as ACR). Control digester is same to the experimental digesters but without the addition of powdered AC (hereafter referred to as CR). Edible oil was used as sole substrate. The working volume of each digester was 0.8 L. All reactors were operated at 35 °C in parallel. The sludge retention time was 30 d. All the experiments were conducted in triplicate.

### Analytical methods

COD were determined using HACH color meter (DR900, USA) according to the manufacturer’s instructions. The pH was recorded using a pH analyzer (Agilent 3200 M, USA). TS and VS were determined based on the weighing method after being dried at 103–105 °C and burnt to ash at 550 °C. The CH_4_ production was determined using a gas chromatograph (Clarus 580 Arnel, PerkinElmer, USA) equipped with a thermal conductivity detector. C, N, S and H elemental analyses in FW were determined using the vario MICRO cube (Elementar, HANAU, Germany). Metals elemental analyses were conducted using an inductively coupled plasma (ICP) – optical emission spectrometer (Perkin Elmer Optima 5300 V, USA). BET surface area and pore volume of activated carbons were measured by N2 adsorption measurement using a Quantachrome Autosorb-6B. Real-time PCR was used to quantify total archaea according to the methods described by Zhang *et al*.^24^.

### Metagenomic shotgun sequencing and metabolic pathways analysis

Sequencing of metagenomic DNA was conducted by IIIumina HiSeq^TM^ sequencer (IIIumina Inc., USA). The analytical methods refer to the reference^25^ that includes DNA extraction, DNA library construction and sequencing, screening of effective reads, assembling of high-quality reads of DNA samples, Gene taxonomic assignment, Gene functional classification, and other related analyses.

Briefly, the metagenomic DNA of the sample was extracted using an extraction kit (MOBIO Laboratories, Inc. Carlsbad, USA) according to the manufacturer’s instructions. The purity of the extracted DNA was checked by determining its absorbance at 260 nm and 280 nm, and we measured the concentration of the DNA using a Qubit 2.0 (life, USA). To obtain the effective and clean sequencing data, raw sequencing results were processed by Trimmomatic^26^ as follows: (1) trimed adaptor sequences of reads; (2) removed sequences containing ambiguities (“Ns”); (3) removed reads shorter than 35nt. (4) removed low - quality sequences i.e. a sequencing quality value lower than 20; (5) removed sequences with the quality of the tail less than 20 bases by sliding window protocol. Effective reads were assembled by IDBA_UD software according to the relationships between reads and overlap to obtain contigs that was further translated into protein sequences^27^. The number of total reads and the basic sequencing statistics of contigs was shown in Table 1. Subsequently, taxonomy was assigned by MetaPhlAn2 software through blasting marker genes with effective reads^28^. Analysis of metabolic pathways and gene functional classification were conducted by DIAMOND^29^ and HUMAnN^30^ through comparing protein sequence with database of Kyoto Encyclopedia of Genes and Genomes (KEGG)^31–35^. Configuration diagram of multiple species/functions including was constructed using Krona^36^ and GraPhlAn^37^. The construction of clustering tree was made according to the hierarchical clustering analysis based on the unweighted pair group method with arithmetic mean. Based on the results of taxonomy, analysis of variance of species was done by STAMP with a screening condition of P value lower than 0.05^38^. Microbial network and co-network were analyzed by QIIME^38^ and SPARCC with dominant species (relative abundance ≥ 1%), respectively.

**Table 1:** The number of reads used for the analysis and the basic sequencing statistics of contigs.

Sample	Total reads	No. of contigs	N50	Max Len	Total Len	Average Len	GC content
A1	76406684	500699	1327	410518	455780585	910.29	50.64%
A2	52543088	421867	1781	286352	465262942	1102.87	51.16%
Seed sludge	62331322	582851	1237	153117	547000062	938.49	55.27%

## Electronic supplementary material

Supplementary Informaiton
