# Supplementary material for: Metagenomic insight into the microbial networks and metabolic mechanism in anaerobic digesters for food waste by incorporating activated carbon
Source: Sci Rep. 2017 Sep 12;7:11293. doi: 10.1038/s41598-017-11826-5 (PMC5595822; doi:10.1038/s41598-017-11826-5)
Supplement: Supplementary file 1 — Supplementary Informaiton [file 41598_2017_11826_MOESM1_ESM.pdf]

THE NATIONAL UNIVERSITY *of* SINGAPORE

Department of Chemical and Biomolecular Engineering

4 Engineering Drive 4, Singapore 117576

Metagenomic insight into the microbial networks and metabolic mechanism in anaerobic digesters for food waste by incorporating activated carbon

Jingxin Zhang[1], Liwei Mao[2], Le Zhang[2], Kai-Chee Loh[2], Yanjun Dai[3], Yen Wah Tong[1,2*]

[1]Environmental Research Institute, National University of Singapore, 1 Create Way, Singapore 138602, Singapore

[2]Department of Chemical & Biomolecular Engineering, National University of Singapore, 4 Engineering Drive 4, Singapore 117576, Singapore

[3]School of Mechanical Engineering, Shanghai Jiao Tong University, 800 Dong Chuan Road, Shanghai 200240, China

Supplementary Material

8 Pages, 11 figures, 1 table

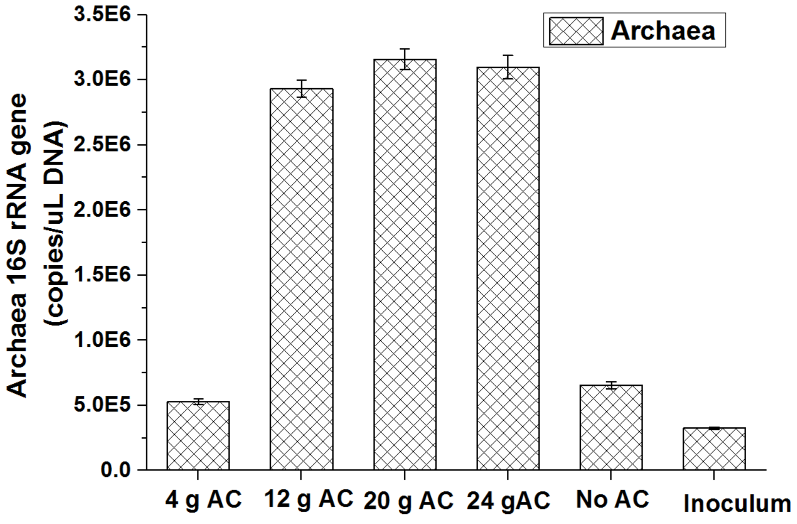

Figure S1. Real-time PCR quantification of total archaea in sludge samples after 51 days of operation.

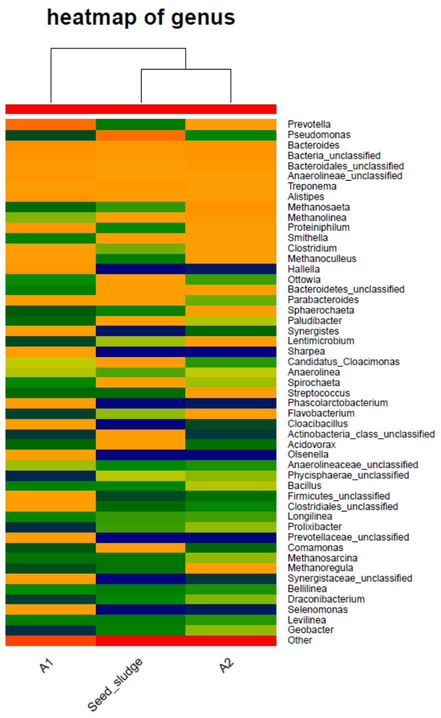

Figure S2. Hierarchical cluster analysis of the whole microbial communities in A1, A2, and seed sludge.

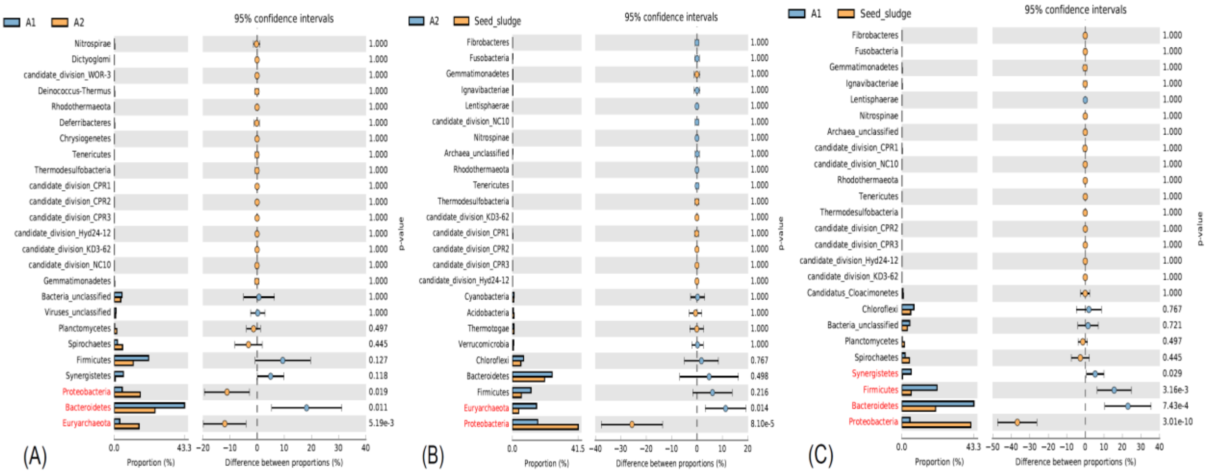

Figure S3. Different proportions of each phylum at 95% confidence intervals between (A) A1 and A2, **(B)** A2 and seed sludge, and **(C)** A1 and seed sludge, respectively.

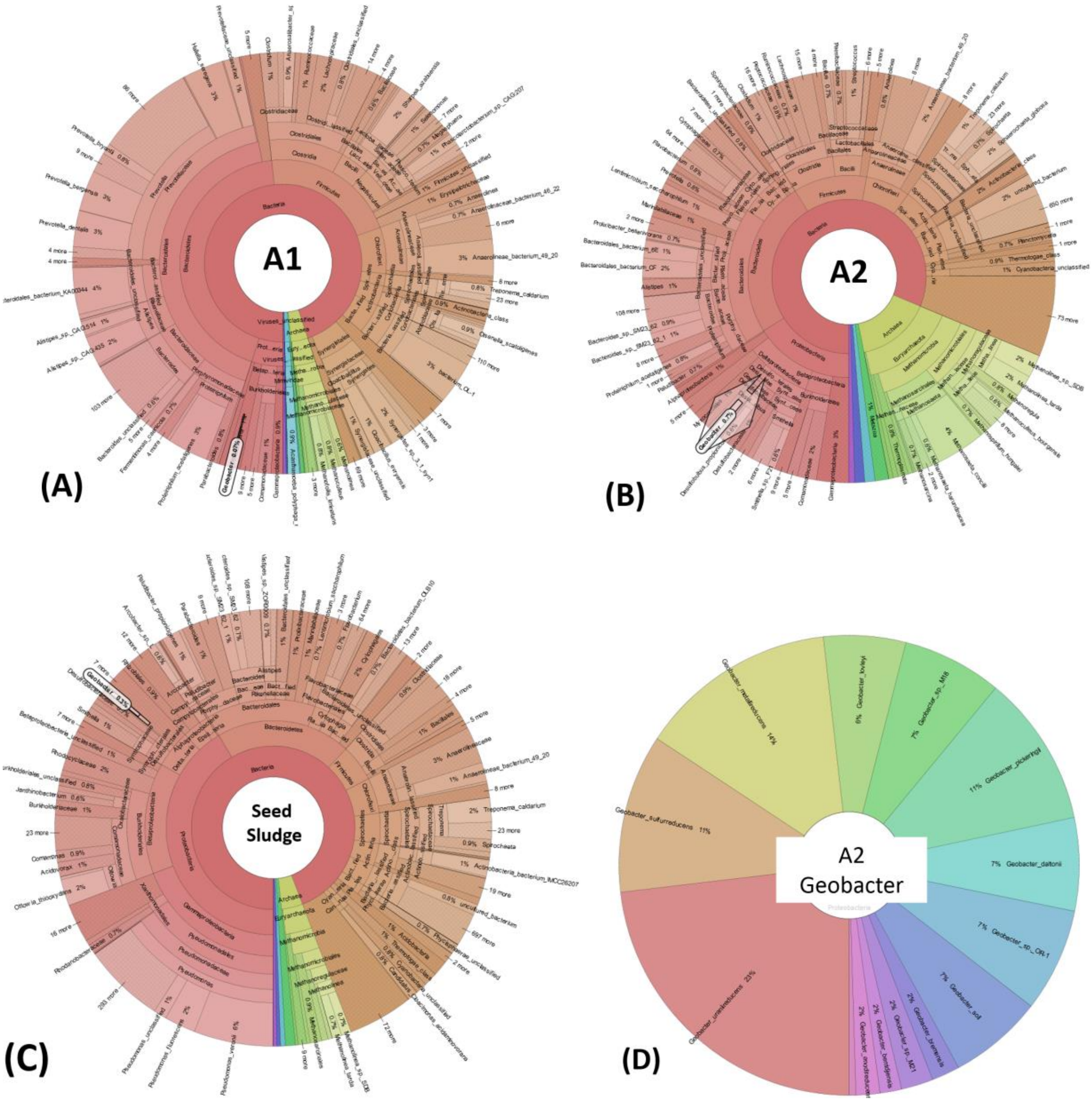

Figure S4. Schematic diagram of multi-level species taxonomy of the whole AD microbial community in the sludge samples of (A) A1, (B) A2, and (C) seed sludge after 51 days of operation. The relative abundance of *Geobacter* was marked in each pie chart. (D) Community compositions of *Geobacter* in reactor A2.

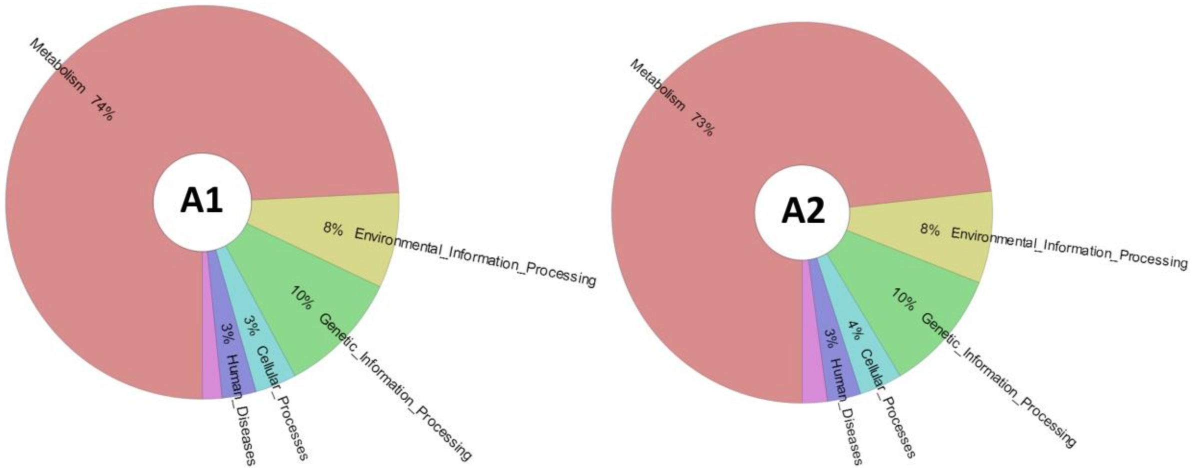

Figure S5. Metabolic pathways of microbial communities, according to gene KEGG pathway analysis. Relative abundance of metabolic pathways in reactor A1 and A2 at different levels.

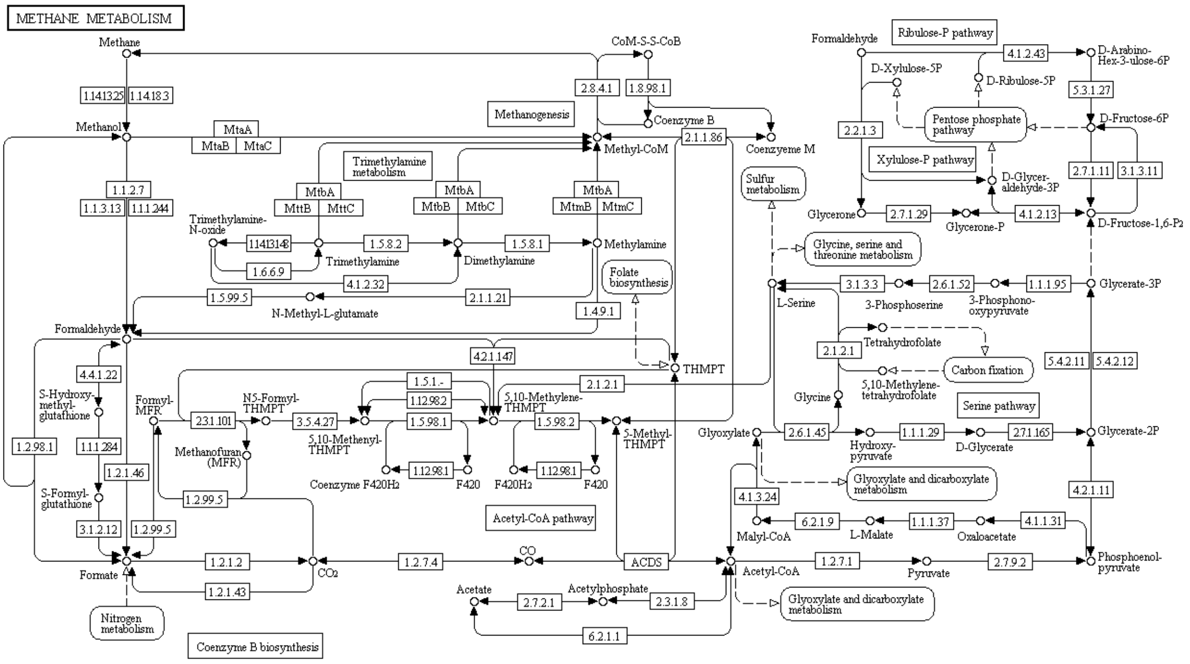

(A)

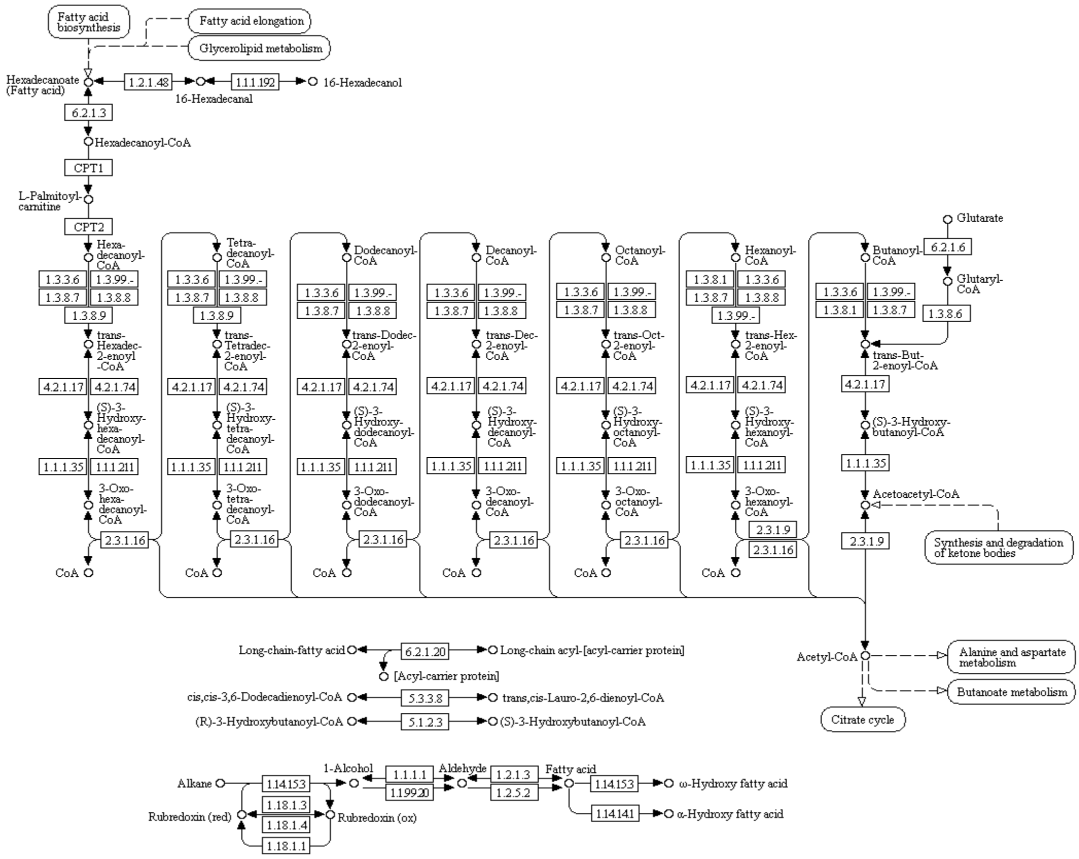

(B)

Figure S6. (A) Pathway of methane metabolism and (B) pathway of fatty acids degradation.[35, 36, 37, 38]

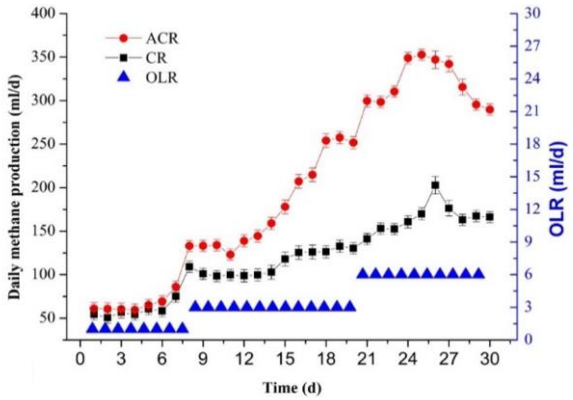

Figure S7. Daily methane production at different loading rate of edible oil in reactors ACR and CR. ACR: a reactor with activated carbon addition. CR: a reactor without activated carbon addition.

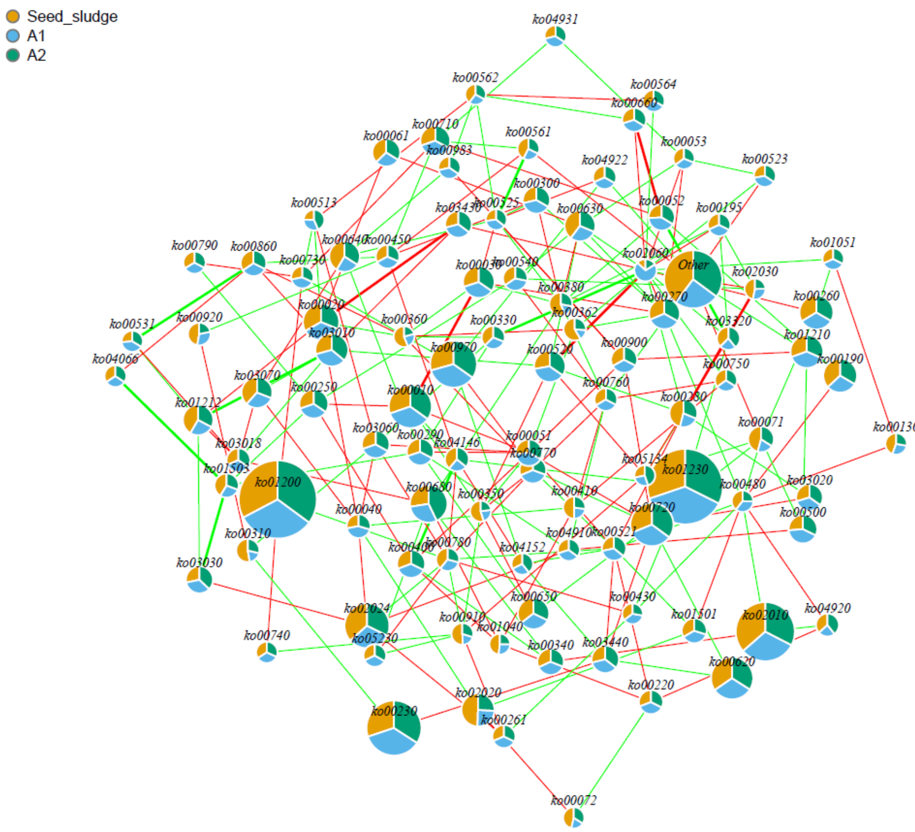

Figure S8. Co-network of metabolic pathways of microbial communities in A1, A2 and seed sludge.

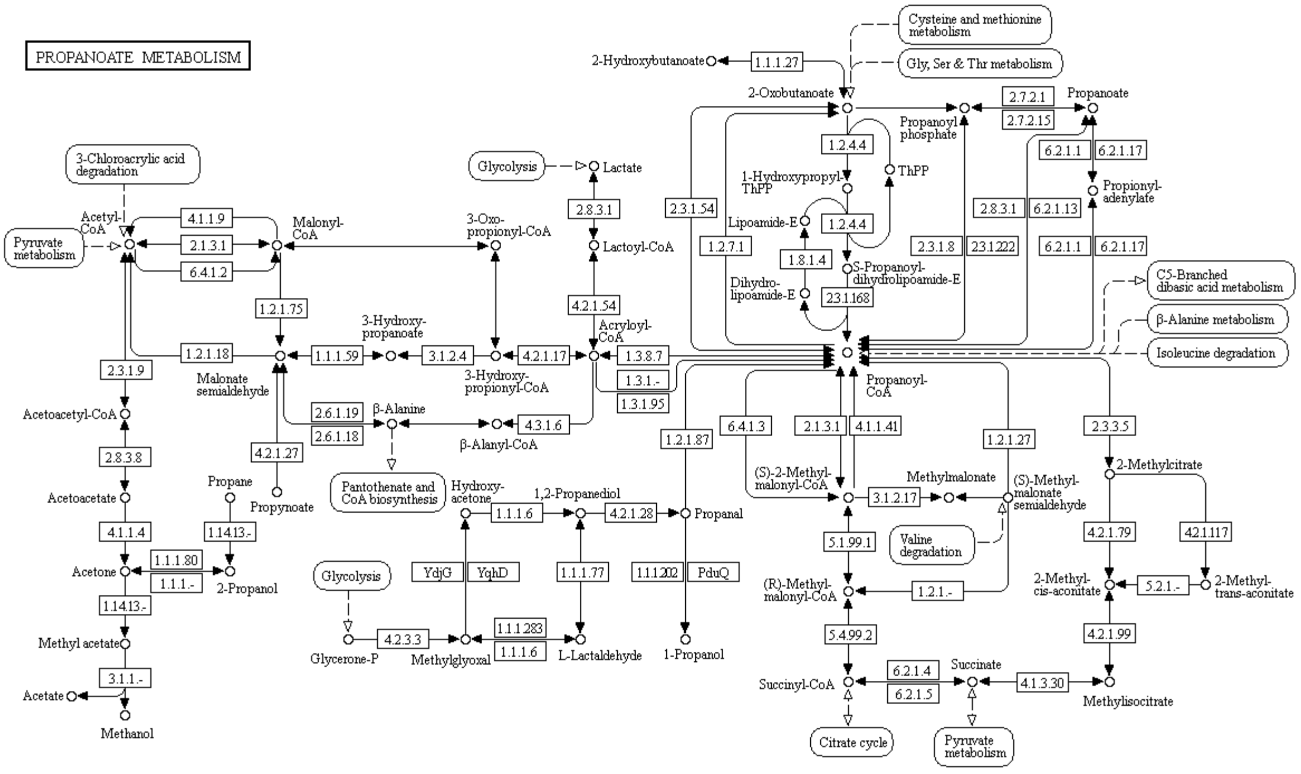

Figure S9. Pathway of propionate metabolism. [35, 36, 37, 38]

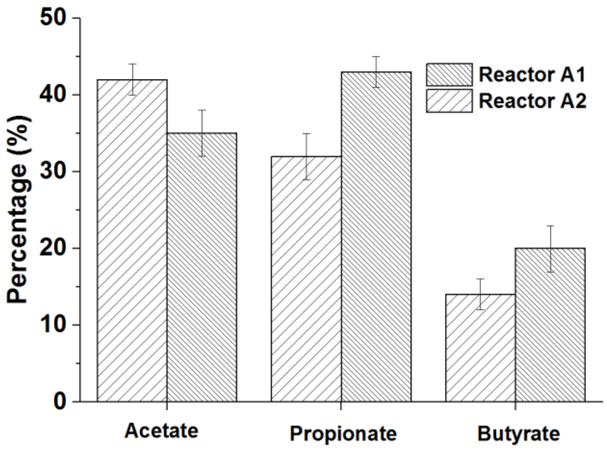

Figure S10. The percentage of main VFA composition of reactor A1 and A2 after 51 days of operation.

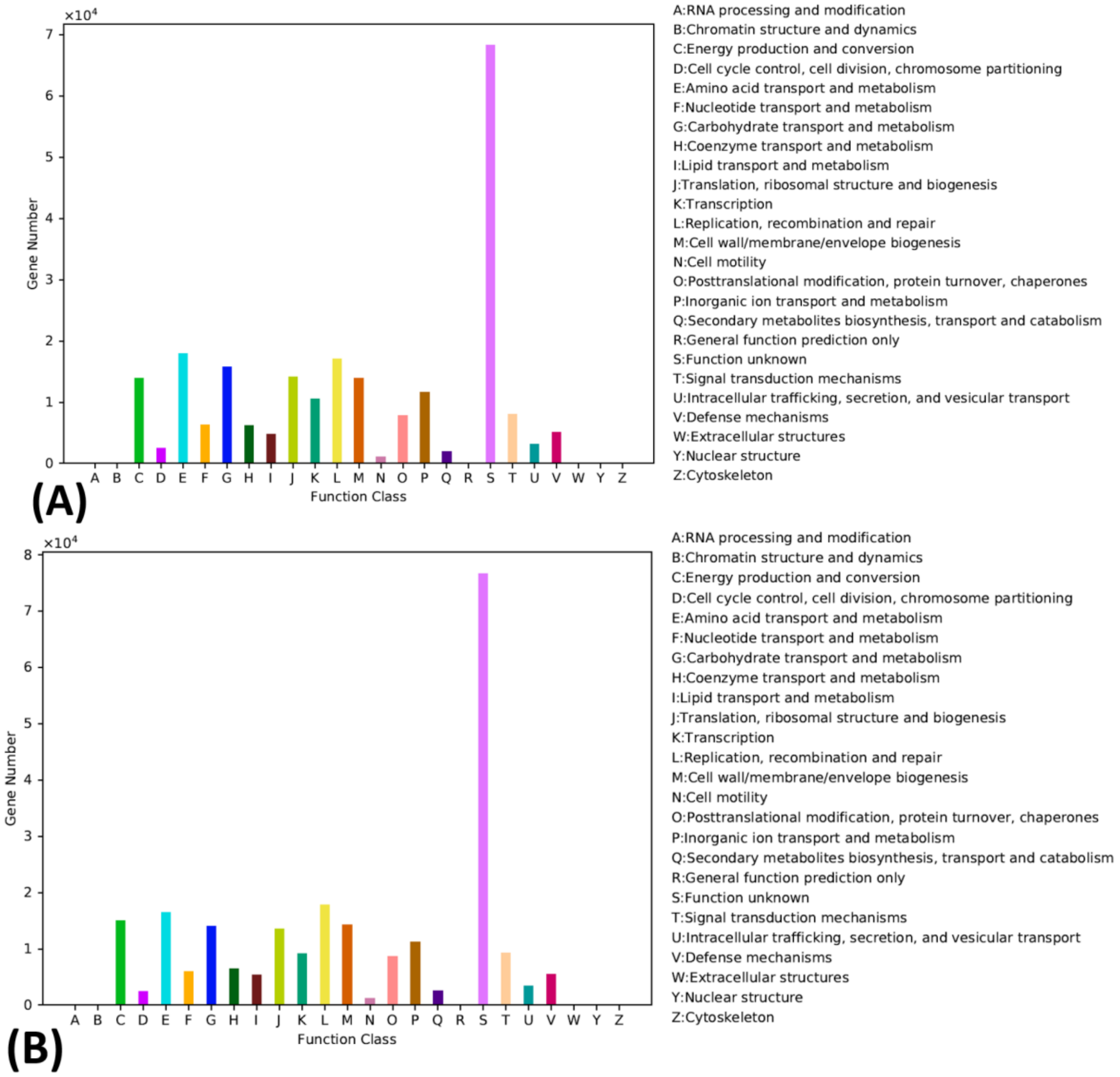

Figure S11. Metabolic pathways of microbial communities, according to gene COG pathway analysis. Gene numbers of metabolic pathways in reactor A1 (A) and A2 (B).

ICP analysis (ppm)										
Na^+	K^+	Ca^{2+}	Mg^{2+}	Fe^{3+}	Cu^{2+}	Zn^{2+}	Al^{3+}	Mn^{2+}	Cr^{3+}	Ni^{2+}
1.42	0.90	2.16	0.14	<0.10	<0.10	<0.10	<0.12	<0.10	<0.10	<0.10
Elemental analysis (wt. %)										
C		N		C/N ratio		S		H		
53.4±0.8		3.7 ± 0.5		14.4		<0.5		7.5 ± 0.1		
Composition analysis										
TS (Wt. %)		VS (Wt. %)								
33.1±0.8		32.3 ± 0.3								

Table S1. Characteristics of food waste. Data are the averages of the values obtained. Error bars represent standard deviations of statistical analysis.
